# Editorial: Mechanisms of drug resistance to targeted therapy in malignancies

**DOI:** 10.3389/fonc.2024.1363808

**Published:** 2024-01-19

**Authors:** Masatsugu Hamaji

**Affiliations:** Department of Thoracic Surgery, Graduate School of Medicine, Kyoto University, Kyoto, Japan

**Keywords:** drug resistance, advanced stage cancer, survival & prognosis, recurrence, mechanism

I, as a thoracic surgeon, am very proud to be one of the guest editors in this Research Topic titled “*Mechanisms of Drug Resistance to Targeted Therapy in Malignancies*” because multimodality treatments contribute to management of cancer patients, including surgery, chemotherapy, radiotherapy, targeted therapy, and immunotherapy. Multimodality management has improved survival of cancer patients. On the other hand, cure from cancer is still challenging, especially in patients undergoing targeted therapy for stage IV or postoperative recurrence (1–3). The reasons include recurrence, metastasis, and acquired drug resistance, which this Research Topic focused on.

This Research Topic published five original studies as follows. Xu et al. addressed anlotinib resistance in osteosarcoma (OS) and explored a new approach to overcome it. Four anlotinib-resistant OS cell lines were established and analyzed through RNA sequencing, identifying IL-6 and STAT3 activation in resistant cells. Tocilizumab, an anti-IL-6 receptor, hindered anlotinib-resistant OS cell progression. Combined tocilizumab and anlotinib treatment inhibited STAT3 expression, curbing tumor growth in cell and mouse models. High IL-6 expression in OS patients correlated with poor prognosis. This suggests that tocilizumab could potentially reverse anlotinib resistance in OS via the IL-6/STAT3 pathway, warranting further investigation for clinical OS treatment.

Chen et al. investigated the use of the neutrophil-to-lymphocyte ratio (NLR) as a predictive biomarker in non-small-cell lung cancer (NSCLC) patients receiving combined chemotherapy and immune checkpoint inhibitors (ICIs). Despite PD-L1’s limited predictive value, previous research indicated that a low NLR predicted survival benefits in high PD-L1 expression cases treated with chemoimmunotherapy. This study focuses on patients with low PD-L1 expression (< 50%) and explores the predictive capacity of NLR. Among 142 patients, those receiving combination therapy had significantly improved progression-free survival (PFS) and overall survival (OS) compared to those on chemotherapy alone. Specifically, in the subgroup with low NLR, combination therapy correlated with significantly extended PFS and OS, confirmed through multivariate analysis. The findings suggest NLR’s potential as a predictive biomarker for identifying survival benefits in advanced NSCLC patients with low PD-L1 expression receiving combined chemotherapy and ICIs.

Breitenecker et al. investigated resistance development in hepatocellular carcinoma (HCC) patients undergoing tyrosine kinase inhibitor (TKI) therapy. Focus lies on Axl receptor tyrosine kinase binding with its ligand Gas6, examining its role in TKI resistance acquisition. Axl-positive and negative HCC cell lines’ initial responses to TKIs were assessed, followed by inducing resistance and conducting RNA-Seq and functional studies to understand molecular mechanisms. Mesenchymal-like phenotypes linked TKI resistance to epithelial plasticity changes. Rego-resistant cells showed elevated Gas6/Axl and ErbB receptor activation. Afatinib, a pan-ErbB inhibitor, reduced viability in Rego-insensitive cells, while genetic manipulation confirmed ErbB2-4 involvement in Rego resistance. Additionally, Rego-resistant cells secreted basic fibroblast growth factor (bFGF) depending on Axl expression. HCC patients treated with Sora or Rego displayed elevated serum bFGF levels, highlighting bFGF as a predictive TKI treatment biomarker. In conclusion, targeting ErbBs in Axl-expressing HCC cells presents a novel vulnerability, suggesting synthetic lethality with Rego and offering insight into potential treatment strategies.

Wang et al. investigated adult T-cell leukemia (ATL) treatment using Lenalidomide (LEN) and a new drug, Iberdomide (IBE). LEN, employed since 2017, lacks fully understood mechanisms and raises concerns about secondary malignancies. The study divides ATL cell lines into LEN-sensitive and -resistant groups, revealing CRBN and IKZF2’s roles in LEN efficacy. DNA analysis highlights distinct gene alterations upon LEN treatment. In mice, oral LEN curbs tumor growth. IBE, a novel cereblon modulator, shows enhanced ATL cell suppression by efficiently degrading IKZF2, suggesting promising therapeutic advantages over LEN for aggressive and relapsed ATL.

Siraj et al. delved into aggressive breast cancer (BC) in Middle Eastern ethnicity, focusing on Polo-like Kinase-1 (PLK1) as a potential therapeutic target. Assessing PLK1 protein expression in over 1500 cases, the research links its overexpression (27.4% of cases) with aggressive clinicopathological features, particularly in triple-negative breast cancer (TNBC) with poor overall survival. Co-expression of PLK1 and PARP, observed in 15.7% of cases, significantly worsened survival compared to either protein alone. In vitro studies demonstrated that combined PLK1 and PARP inhibition reduced cell survival and induced apoptosis in TNBC cell lines, suggesting effectiveness even in PARP inhibitor-resistant cases. These findings emphasize PLK1’s role in Middle Eastern BC, advocating for combined PLK1 and PARP inhibition as a potential treatment strategy.

As described above, this Research Topic should be noted for including both basic and clinical studies of various malignancies. All the above contributions are seminal work that would improve in the near future, survival of patients undergoing targeted therapy for advanced-stage cancers.

## Author contributions

MH: Writing – original draft, Writing – review & editing.
